# 2042. Impact of the COVID-19 Pandemic on Healthcare-Associated Infections (HAI) by Race and Ethnicity in a Large Network of Community Hospitals: A Call to Action

**DOI:** 10.1093/ofid/ofac492.1664

**Published:** 2022-12-15

**Authors:** Erin Gettler, Ibukunoluwa Kalu, Sonali D Advani, Jessica Seidelman, Jay Raj Krishnan, Melissa Campbell, Sarah S Lewis, Becky A Smith, Deverick J Anderson

**Affiliations:** Duke University Medical Center, Durham, North Carolina; Duke University Medical Center, Durham, North Carolina; Duke University School of Medicine, Durham, North Carolina; Duke University School of Medicine, Durham, North Carolina; Duke University Medical Center, Durham, North Carolina; Duke University Medical Center, Durham, North Carolina; Duke University Medical Center, Durham, North Carolina; Duke University, durham, North Carolina; Duke University, durham, North Carolina

## Abstract

**Background:**

Inequities in healthcare among racial and ethnic minorities are globally recognized. The focus has centered on access to healthcare, equitable treatment, and optimizing outcomes. However, there has been relatively little investigation into potential racial and ethnic disparities in HAI.

**Methods:**

We performed a retrospective cohort analysis of select HAI prospectively-collected by a network of community hospitals in the southeastern US, including central line-associated bloodstream infection (CLABSI), catheter-associated urinary tract infection (CAUTI), and laboratory-identified *Clostridioides difficile* infection (CDI). Outcomes were stratified by race/ethnicity as captured in the electronic medical record. We defined the pre-pandemic period from 1/1/2019 to 2/29/2020 and the pandemic period from 3/1/2020 to 6/30/2021. Outcomes were reported by race/ethnicity as a proportion of the total events. Relative rates were compared using Poisson regression.

**Results:**

Overall, relatively few facilities consistently collect race/ethnicity information in surveillance databases within this hospital network (< 40%). Among 21 reporting hospitals, a greater proportion of CLABSI occurred in Black patients relative to White patients in both study periods (pre-pandemic, 49% vs 38%; during pandemic, 47% vs 31%; respectively, **Figure 1a**), while a higher proportion of CAUTI and CDI occurred in White patients (**Figures 1b-c**). Black patients had a 30% higher likelihood of CLABSI than White patients in the pre-COVID period (RR, 1.30; 95% CI, 0.83-2.05), which was not statistically significant (**Table 1**). However, this risk significantly increased to 51% after the start of the pandemic (RR, 1.51; 95% CI, 1.02-2.24). Similar trends were not observed in other HAI (**Tables 2-3**).

**Figure:**
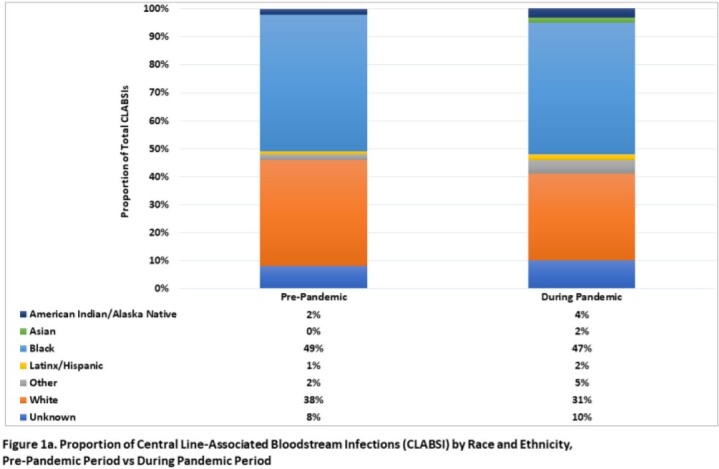

**Figure:**
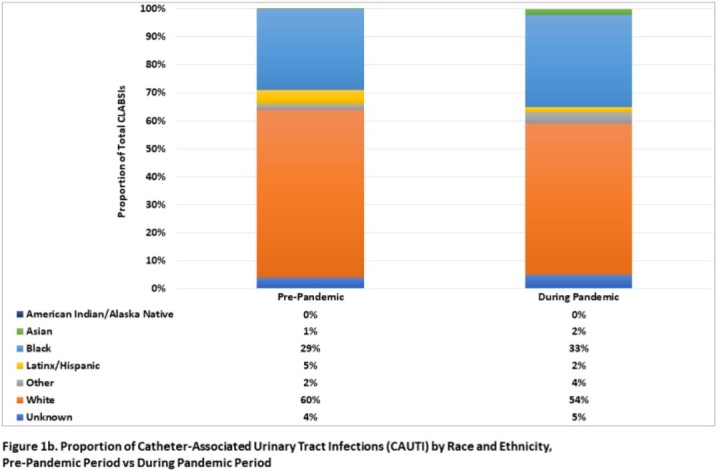

**Figure:**
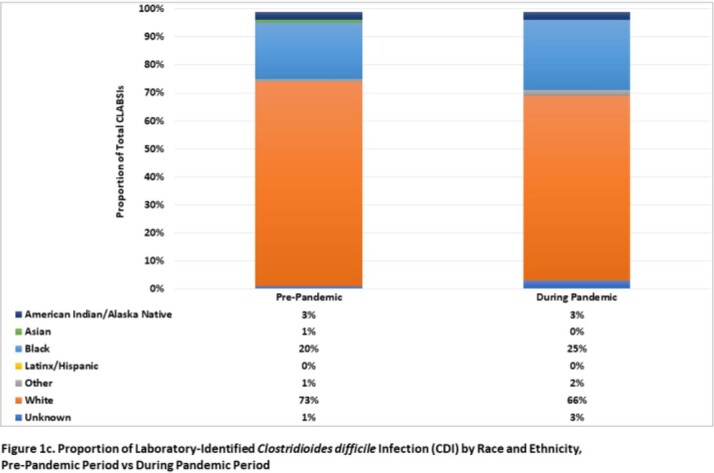

**Conclusion:**

We found differences in HAI rates by race/ethnicity in a network of community hospitals. Black patients had higher likelihood of CLABSI, and this likelihood increased during the pandemic. Patient safety events, including HAI, may differ across racial and ethnic groups and negatively impact health outcomes.

**Figure:**
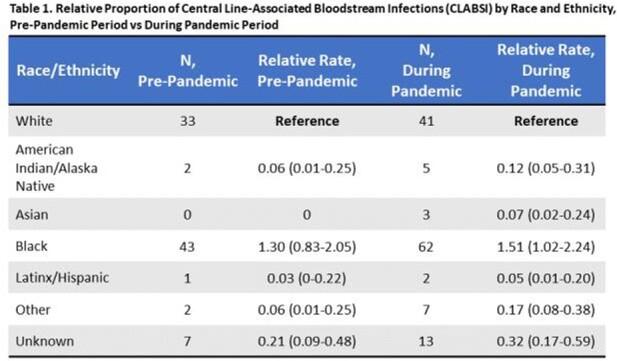

**Figure:**
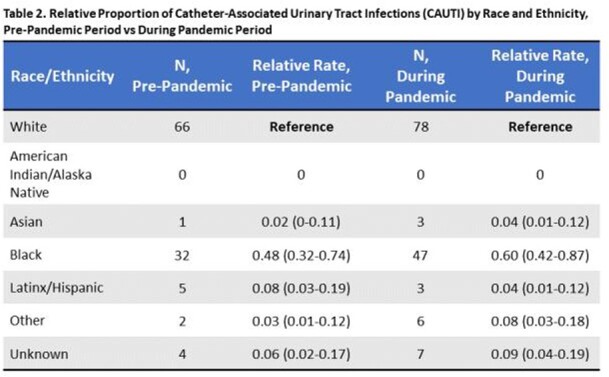

**Figure:**
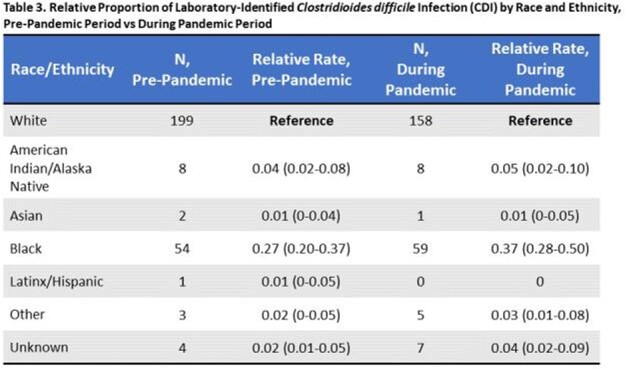

**Disclosures:**

**Sonali D. Advani, MBBS, MPH, FIDSA**, Locus Biosciences: Advisor/Consultant|Locus Biosciences: Honoraria|Sysmex America: Advisor/Consultant.

